# Dp71 depleted HBE cells displayed increased DNA damage and apoptosis induced by H_2_O_2_

**DOI:** 10.1186/s11658-019-0169-6

**Published:** 2019-06-17

**Authors:** Sichuang Tan, Shuai Zhao, Xuefei Xiao, Lan Xiao, Jinliang Xie, Sipin Tan

**Affiliations:** 1 Key Laboratory of Sepsis Translational Medicine of Hunan, Department of Pathophysiology, Xiangya School of Medicine, Central South University, Changsha, Hunan Province 410008 People’s Republic of China; 2 Department of Thoracic Surgery, the Second Xiangya Hospital, Central South University, 139 Ren-min Road, Changsha, Hunan Province 410011 People’s Republic of China; 3 Center of Transplant Surgery, Xiangya Hospital, Central South University, Changsha, Hunan Province 410008 People’s Republic of China; 4 Department of Traditional Chinese Medicine, the Third Xiangya Hospital, Central South University, Changsha, Hunan Province People’s Republic of China; 5 Department of Emergency and Critical Care Medicine, the Third Xiangya Hospital, Central South University, Changsha, Hunan Province People’s Republic of China; 6 Department of Pathology, Tianjin Medical University Cancer Institute and Hospital, Tianjin, 300060 People’s Republic of China

**Keywords:** Dp71, DNA damage, Apoptosis, RAD51, FAK

## Abstract

Human bronchial epithelium (HBE)-Dp71 anti-sense(AS)cells with stably transfected Dp71 siRNA plasmids were prepared for further exploration of Dp71 biological traits in cells other than PC12. HBE-Dp71AS cells displayed increased DNA damage induced by H_2_O_2_. Apoptosis of HBE-Dp71AS cells induced by H_2_O_2_ was increased via enhancing caspase 3, caspase 8 and caspase 9. HBE-Dp71AS cells also displayed decreased proliferation and clonogenic formation. RAD51 was proved to be a new binding partner of Dp71 by co-immunoprecipitation (Ip) and immunofluorescence. Reduced RAD51 mRNA and protein levels were observed in HBE-Dp71AS cells. Decreased lamin B1, focal adhesion kinase (FAK), phosphorylated focal adhesion kinase (p-FAK) and phosphorylated protein kinase B (p-AKT) were detected in the HBE-Dp71AS cells, which functioned together with RAD51 as the molecular explanations for the character alterations of HBE-Dp71AS cells.

**Electronic supplementary material:**

The online version of this article (10.1186/s11658-019-0169-6) contains supplementary material, which is available to authorized users.

## Introduction

Dystrophin Dp71 is one of the most widely expressed isoforms of dystrophin, the pathogenic gene of Duchenne muscular dystrophy (DMD), an X-linked recessive disorder [1]. Functioning as one of the most ubiquitously expressed isoforms of dystrophin, Dp71 is a 70- to 75-kDa protein located in all tissues except skeletal muscle [2, 3]. Previous research on Dp71 identified its crucial role for cell adhesion, neuronal differentiation and the cell cycle in PC12 cells. Dp71 was proved to be a putative tumor suppressive gene in gastric cancer [4–6]. Our preliminary clinical work also identified reduced Dp71 expression in lung cancer. Considering HBE as a usual cell model for pulmonary functional analysis, a shRNA strategy was used to knock down Dp71 in HBE to further clarify its biological significance. HBE-AS cells displayed increased DNA damage under oxidative stress, and decreased proliferation and clone formation capabilities. In a caspase-dependent way, HBE-AS cells displayed an increased apoptosis rate induced by H_2_O_2_. Our further characterization of HBE-AS cells identified RAD51, lamin B1, FAK and AKT to be the molecular explanations for the altered phenotypes of HBE-AS cells.

## Material and methods

### Construction of Dp71 short hairpin RNA plasmid

According to the open reading frame of the human Dp71 gene (NM_004015), one siRNA sequence (5′-gcactttaattatgacatc-3′) was selected. The scrambled sequence (5′-ttctccgaacgtgtcacgt-3′) which has no significant homology with human gene sequences was included as a negative control. Two complementary oligonucleotides for Dp71 (5′-gatcccgt**ctttagctgacctgaataa***ctcgag***ttattcaggtcagctaaagac**tttttggat-3′ and 5′-agctatccaaaaagt**ctttagctgacctgaataa***ctcgag***ttattcaggtcagctaaagac**gg-3′), and for the negative control (5′-gatccc**ttctccgaacgtgtcacgt***ctcgag***acgtgacacgttcggagaa**tttttggat-3′ and 5′-agctatccaaaaa**ttctccgaacgtgtcacgt*ctc****gag*a**cgtgacacgttcggagaa**gg-3′), were synthesized by Invitrogen. Sense or antisense strands are in bold letters and stem loop sequences are in italics. They were annealed to generate double-stranded DNAs and ligated into the linearized shRNA (short hairpin RNA) eukaryotic expression vectors purchased from Genechem (Shanghai, China, containing hU6-MCS-CMV-GFP-SV40-Neomycin elements) to construct Dp71 shRNA or control empty shRNA vectors, which were termed Dp71AS and Dp71 empty shRNA vector (E) respectively. The nucleotide sequences of the plasmids were verified by automated DNA sequencing.

### Cell culture and generation of stable transfectants

HBE was obtained from the Culture Center, Chinese Academy of Medical Sciences (Shanghai, China). HBE cells were cultured in the same condition as described previously [7]. For stable transfectants, 5 μg of Dp71shRNA plasmid or 5 μg of control empty shRNA plasmid was mixed with 15 μl of Lipofectamine in serum- and antibiotics-free 1640, and the DNA/Lipofectamine mixture was added to the cell culture medium and incubated in the incubator for 4 h. The transfection mixture was removed and cells were maintained in 1640 supplemented with sera. Selection of stable transfectants was initiated with 600 μg/ml of G418 (Invitrogen) 48 h after transfection, a neomycin analog. The stable transfected HBE cells were named HBE-Dp71AS and HBE-Dp71E respectively.

### Isolation of cell extracts and western blot analysis

Cultured cells were collected by centrifugation at 1200 rpm for 5 min, and washed twice with PBS. Protein extraction, concentration determination, 10% SDS-PAGE electrophoresis, and membrane incubation with the corresponding primary antibody (rabbit anti-dystrophin, rabbit anti-RAD51 polyclonal antibody purchased from Abcam; rabbit anti-FAK polyclonal antibody, p-FAK polyclonal antibody; rabbit anti-Akt polyclonal antibody, p-Akt polyclonal antibody; rabbit anti-phospho-histone H2AX (γH2AX; Ser 139) antibody (Bioworld Technology, Inc) was performed as described previously. After three washes with TBS-T, horseradish peroxidase-conjugated anti-rabbit IgG was used as the secondary antibody and developed using the ECL Western blotting analysis system (Amersham-Pharmacia).

### Quantitative real-time polymerase chain reaction (QRT-PCR) and RT-PCR

The following primers were used and they produced a 157 bp PCR product for Dp71: 173 bp PCR product for FAK, 146 bp PCR product for lamin B1, 160 bp PCR products for RAD51 and 181 bp PCR product for 18 s. The primers are: LMNB1 (Human Accession NM_005573) F:5′tccaggagaaggaggagctg3′, R:5′ggtctcgtagagcgccttg3′; Dp71 (Human Accession NM_004017.2) F:5′ttggcagtcaaacttcggactc3′,R:5′gtgtcctctctcattggctttccag3′; FAK (Human Accession L13616.1) F: 5′ tccccagagctcctcaagaa 3′,R: 5′ tactcgctccattgcaccag3′; RAD51 (Human Accession D14134.1) F: 5′gggaagacccagatctgtca 3′, R: 5′catcactgccagagagacca 3′; Human 18S (NM_022551) F: 5′ aaatagcctttgccatcactgcc3′,R: 5′ gttcaagaaccagtctgggatc3′.

### Cell viability assay

The cell viability was assessed by conducting the 3-(4,5-dimethylthiazol-2-yl)-2,5-diphenyltetrazolium bromide (MTT) assay. MTT assay and results interpretations were performed as described previously [8].

### Plate colony formation assay

Clone formation assay was performed as described previously. Clone formation efficiency was calculated according to the formula: (clone number/plated cell number) × 100% [7].

### Apoptosis assay

Apoptosis of HBE, HBE-Dp71AS and HBE-Dp71E cells in the log growth phase were induced by 0.2 mM H_2_O_2_ (Sigma, St. Louis, USA) for 16 h. The cells then were harvested by trypsinization for flow cytometry. Apoptosis was quantified using the PE Annexin V apoptosis detection kit (BD Pharmingen, San Diego, USA) according to the manufacturer^’^s protocol. Cell analyses were made using a FACSCalibur flow cytometer (Becton-Dickinson, Mountain View, CA) and CellQuest software (BD Biosciences). Each assay was repeated 3 times [7].

### Measurement of caspase 3, 8, 9 activities

The caspase fluorescent assay kits specific for caspase 3, caspase 8 and caspase 9 (BioVision, San Francisco, USA) were used to detect caspase activation by measuring the cleavage of a synthetic fluorescent substrate. Cell treatment and fold increases in caspase 3, caspase 8 and caspase 9 activities were determined as described previously [8].

### Alkaline comet assay for DNA damage

To perform the comet assay, the cell suspension of each cell group was mixed with low melting-point agarose at 37 °C, to a final concentration of 0.7%. The mixture (15 μl) was pipetted onto slides pretreated with 0.5% normal-melting-point agarose, to retain the agarose cell suspension. The drop containing the cells was covered with a glass cover slip (24 mm × 24 mm) and left at 4 °C for 5 min. The cover slips were gently removed and the slides were then ready for processing. The alkaline comet assay was performed using the basic rationale of Singh et al. The slides were then incubated in the dark for 30 min in cold electrophoresis buffer (300 mM NaOH, 1 mM EDTA, 1% (v/v) DMSO, pH 13) to allow the DNA to unwind before electrophoresis at 25 V for 25 min. After neutralization with 0.5 M Tris–HCl (pH 8.0), the slides were stained with 50 μl ethidium bromide (30 μg/mL, Absin Bioscience Inc., China). Finally, the images were taken by fluorescence microscope and at least 120 randomly selected cells (30 cells from each of the three replicate slides) were analyzed per sample and analyzed using the Comet Assay Software Pect (CASP 1.2.3 beta 1) (http://casplab.com/download). Parameters of tail moment (% DNA in tail × tail length), tail length, and percent of DNA in tail, the most frequently used parameters in the comet assay, were used in this study.

### Immunofluorescence and confocal microscopy analysis

The immunofluorescence and confocal microscopy analysis of Dp71, Rad51 and γ-H2AX in HBE was as follows: After the three HBE cells were cultured on glass coverslips for 24 h, cells were treated with 200 μM H_2_O_2_ for 30 min as described previously, treated cells and untreated cells were incubated overnight at 4 °C with the primary anti-dystrophin, anti-RAD51 and anti-γ-H2AX antibody. Cells were incubated for 10 min at 37 °C with 1 mg/ml 49,6-diamidino-2-phenylindole (DAPI) for counterstaining, After washing, coverslips were mounted on microscope slides with VectaShield (Vector Laboratories, Inc., Burlingame, CA, USA) and analyzed in a confocal and multiphoton microscope (TCS-SP5, Leica Microsystems, Heidelberg, Germany), using an oil immersion 636 objective. Co-localization of FITC, TRITC, and DAPI staining was analyzed in single optical sections obtained for two channels throughout the Z axis.

### Immunoprecipitation

Total protein extracts in a final volume of 250 ml were incubated overnight at 4 °C with 5 μg of rabbit anti-lamin B1, 5 μg of rabbit anti-Dp71 antibody, 5 μg of rabbit anti-FAK, and 5 μg of rabbit anti-RAD51 antibody, previously bound to protein G magnetic beads (Millipore). An irrelevant rabbit polyclonal antibody bound to protein G magnetic beads was performed as a negative control. The immune complexes were precipitated by placing the tube into the magnetic stand (Millipore) and washing 3 times with 500 μl of PBS containing 0.1% Tween 20. Precipitated proteins were separated by SDS-PAGE and analyzed by Western blotting with mouse anti-lamin B1, mouse anti-Dp71 antibody, mouse anti-RAD51 and mouse anti-FAK antibody.

### Statistical analyses

All assays were repeated 3 times to ensure reproducibility. Results were displayed as mean ± SE. One-way ANOVA and LSD were used to analyze all experimental data. All statistical analyses were performed with SPSS software (version 17.0; SPSS Inc., Chicago, IL, USA). *P* < 0.05 was considered as indicating a statistically significant difference.

## Results

### Establishment of Dp71 depleted HBE cell lines

**Fig. 1:**
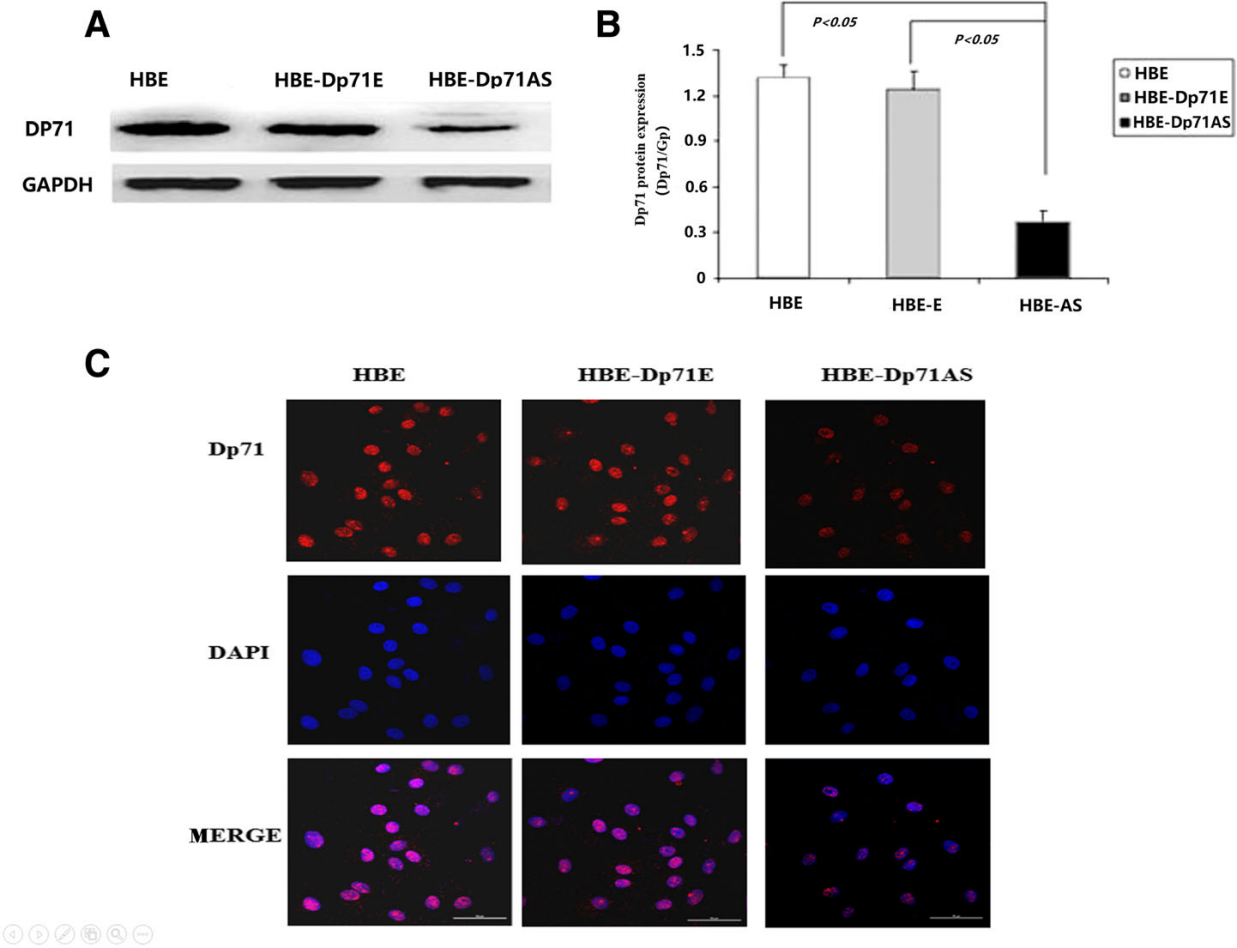
Dp71 expression in HBE, HBE-Dp71E and HBE-Dp71AS cells. **a** The Dp71 protein was reduced by 70% in HBE-Dp71AS cells compared with that in the control cells. Statistical analysis. **b** revealed that the differences are significant. **c** Immunofluorescence proved the significant reduction of Dp71 in HBE-Dp71AS cells (Scale bar: 50 μm)

After 4 weeks’ selection, G418-resistant HBE cells were obtained. According to the short hairpin RNA plasmids transfected, the cells were named HBE-Dp71AS and HBE-Dp71E. Western blotting showed that the protein expression of Dp71 was markedly downregulated by 70% in cells transfected with the Dp71 shRNA construct (Fig. 1a), while transfection of the control empty vector had little effect on Dp71 expression. These results suggested that Dp71 shRNA constructs potently and specifically inhibited endogenous Dp71 protein expression in HBE cells, and the differences were statistically significant (Fig. 1b). Immunofluorescence also clearly showed reduced Dp71 expression in HBE-Dp71AS cells (Fig. 1c). The stable HBE-Dp71AS and HBE-Dp71E cell lines were used for further functional analyses.

### HBE-Dp71 AS cells displayed increased DNA damage induced by H_2_O_2_

**Fig. 2:**
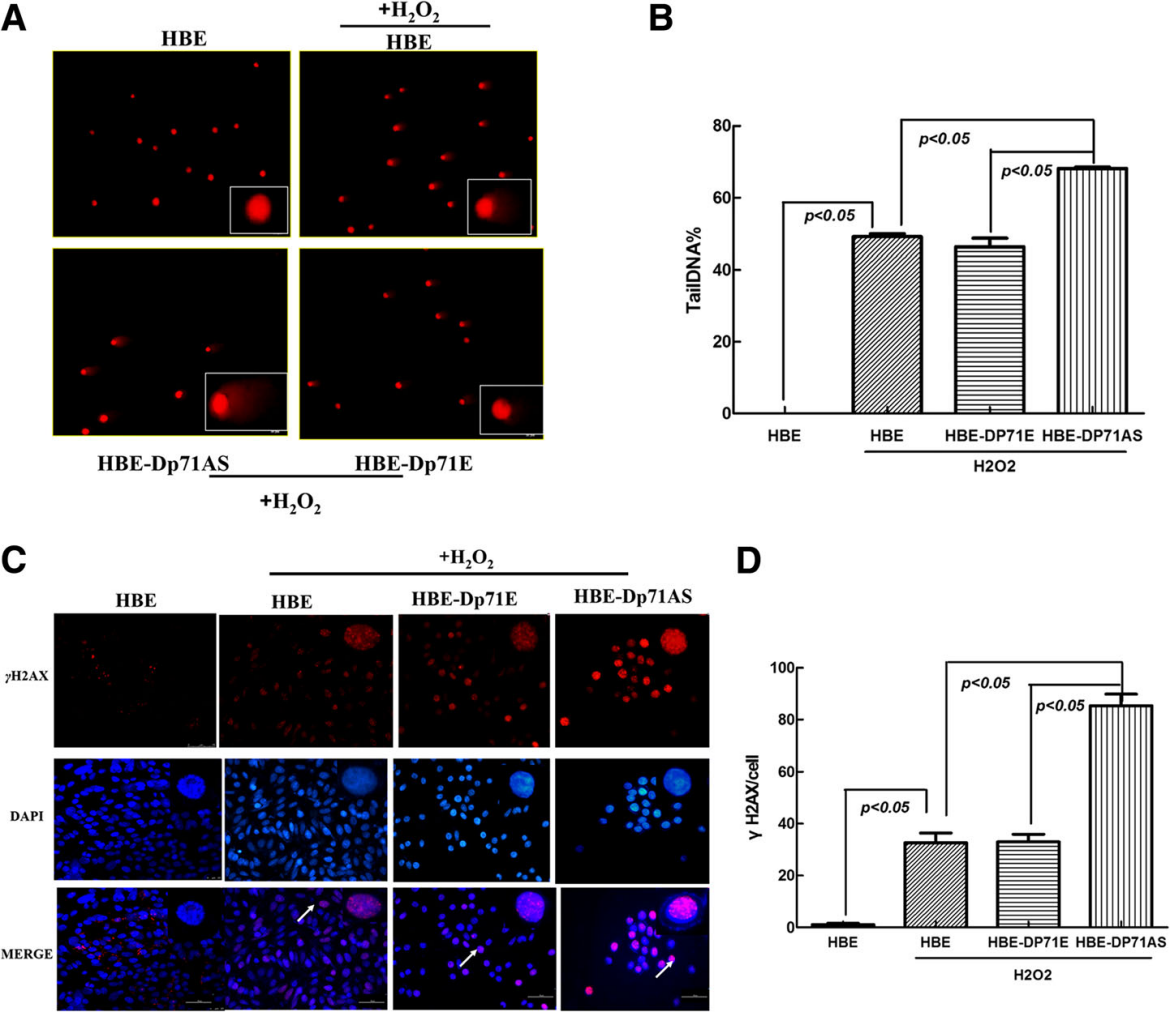
HBE-Dp71 AS cells displayed increased DNA damage induced by H_2_O_2_. **a** Images of comets obtained by alkaline comet assay representing different degrees of DNA damage of HBE cell lines. **b** Statistical analysis of %Tail DNA measured in three HBE cell lines using CometScore software proved that the differences were significant. **c** γ-H2AX nucleus foci formation in HBE cell lines. After the three HBE cells were treated by 0.2 mM H_2_O_2_, more γ-H2AX nuclear foci were formed in HBE-Dp71 AS cells (scale bar: 50 μm). **d** Statistical analysis showed that the differences between the γH2AX foci formed per cell induced by H_2_O_2_ were significant

After the HBE, HBE-Dp71E and HBE-Dp71AS cells had been exposed to 0.2 mM H_2_O_2_ for 30 min, the comet assay was used to analyze single strand breaks in these three HBE cell lines. In normal cells, the fluorescence is confined mostly to the nucleus because undamaged DNA cannot migrate. In cells with DNA damage, DNA is denatured by the alkaline solution used for single-strand break detection. The negatively charged DNA fragments are then released from the nucleus and migrate toward the anode. Compared with HBE and HBE-Dp71E cells, HBE-Dp71 AS cells displayed more serious DNA damage (Fig. 2a and b). Then the foci numbers of histone H2AX phosphorylated at serine 139 (γ-H2AX) were examined for detection of DSB (double strand breaks). As indicated in Fig. 2c, clearly more foci of γ-H2AX were detected in HBE-Dp71 AS cells, compared with HBE, and Dp71E cells. Statistical analysis (Fig. 2d) proved that the differences were significant. Alkaline comet assay and γ-H2AX analysis showed enhanced DNA damage induced by H_2_O_2_ in HBE-Dp71 AS cells.

### Dp71 knockdown HBE cells displayed increased H_2_O_2_-induced apoptosis via enhanced caspase 3, caspase 8 and caspase 9 activation

**Fig. 3:**
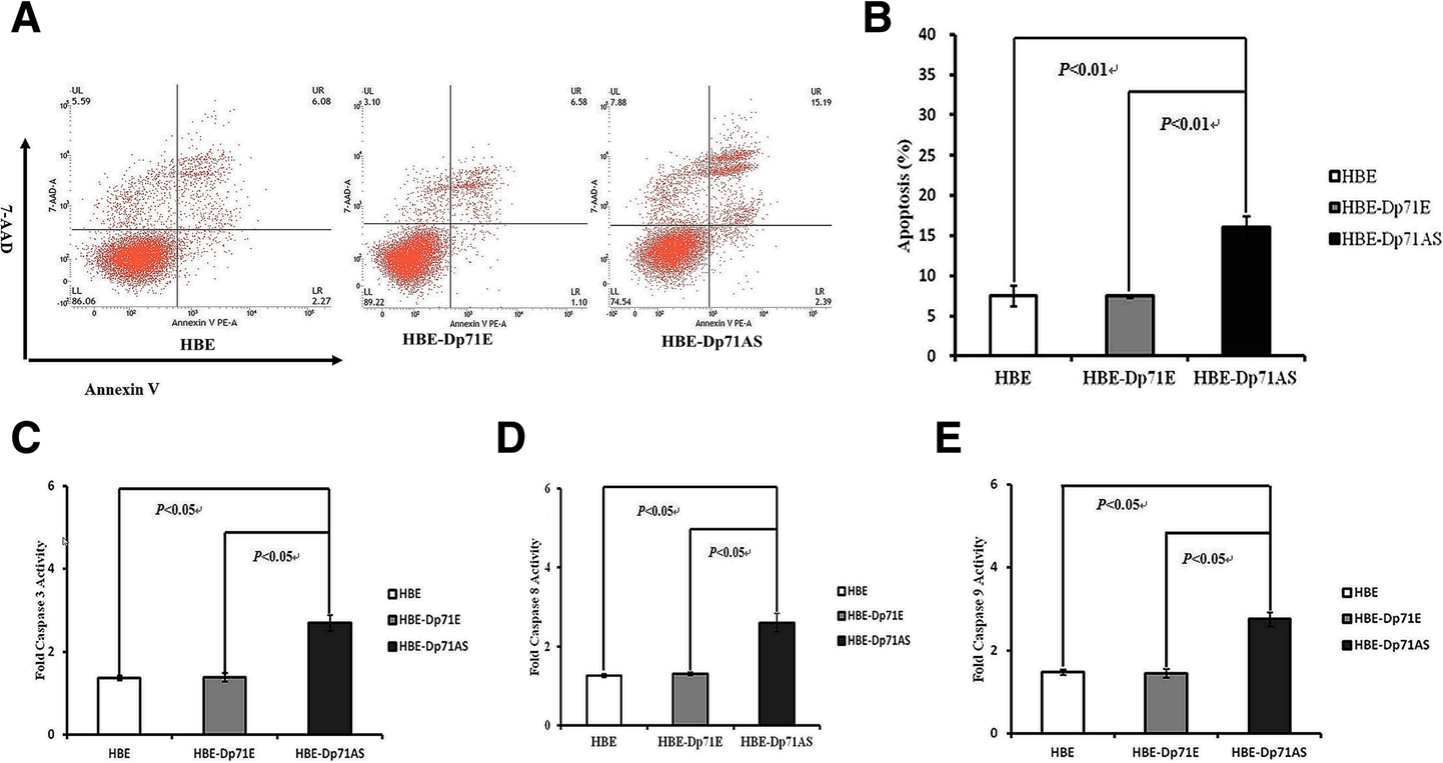
HBE-Dp71AS cells displayed enhanced H_2_O_2_ induced apoptosis via increased activation of caspase 3, 8 and 9. **a** Three representative dot plots of the HBE-Dp71AS cells, HBE and HBE-Dp71E cells exposed to H_2_O_2_ treatment. Apoptosis was determined by Annexin V/PE staining and flow cytometric analysis. Similar results were observed from 3 independent experiments. **b** Statistical analysis of the apoptosis induced by H_2_O_2_ of the HBE-Dp71AS cells, HBE and HBE-Dp71E cells. **c** caspase 3, (**d**) caspase 8 and (**e**) caspase 9 activity measurement

Cytoskeleton protein is one of the key intracellular components resisting the oxidative stress induced injury which occurs in many circumstances such as ischemia and hypoxia. In order to find out whether the reduction of Dp71 can alter the antioxidant defense capabilities of HBE cells, apoptosis rates of the three HBE cell lines induced by H_2_O_2_ (0.2 mM) for 16 h were analyzed. The apoptosis rates for HBE-Dp71AS, HBE-Dp71E and HBE were 16.00 ± 1.43, 7.47 ± 0.19 and 7.51% ± 1.27% respectively after the H_2_O_2_ stimulation. As shown in dot plot images generated by FACS analysis of cells stained with PE Annexin V (Fig. 3a), 8% more apoptosis was detected in HBE-Dp71AS cells compared with the HBE-Dp71E and HBE cells; the differences were statistically significant (Fig. 3b).

H_2_O_2_ can induce the apoptosis via activation of both the extrinsic death receptor apoptosis pathway and the intrinsic apoptotic pathway. Caspase 9 is an initiator of the intrinsic pathway of apoptosis; caspase 8 is an indispensable enzyme of the extrinsic pathway. The activation of both caspase 8 and 9 will ultimately result in the activation of the effector caspase 3. As shown in Fig. 3c, significant fold change of caspase 3 in HBE-Dp71AS was observed compared with HBE and HBE-Dp71E cells after they had been treated with H_2_O_2._ Significant fold changes of caspase 8 and 9 are shown in Fig. 3d and e. To sum up, the ablation of Dp71 increased the H_2_O_2_-induced apoptosis via enhancing the activations of caspase 3, 8 and 9.

### Dp71 knockdown HBE cells displayed decreased proliferation rate

**Fig. 4:**
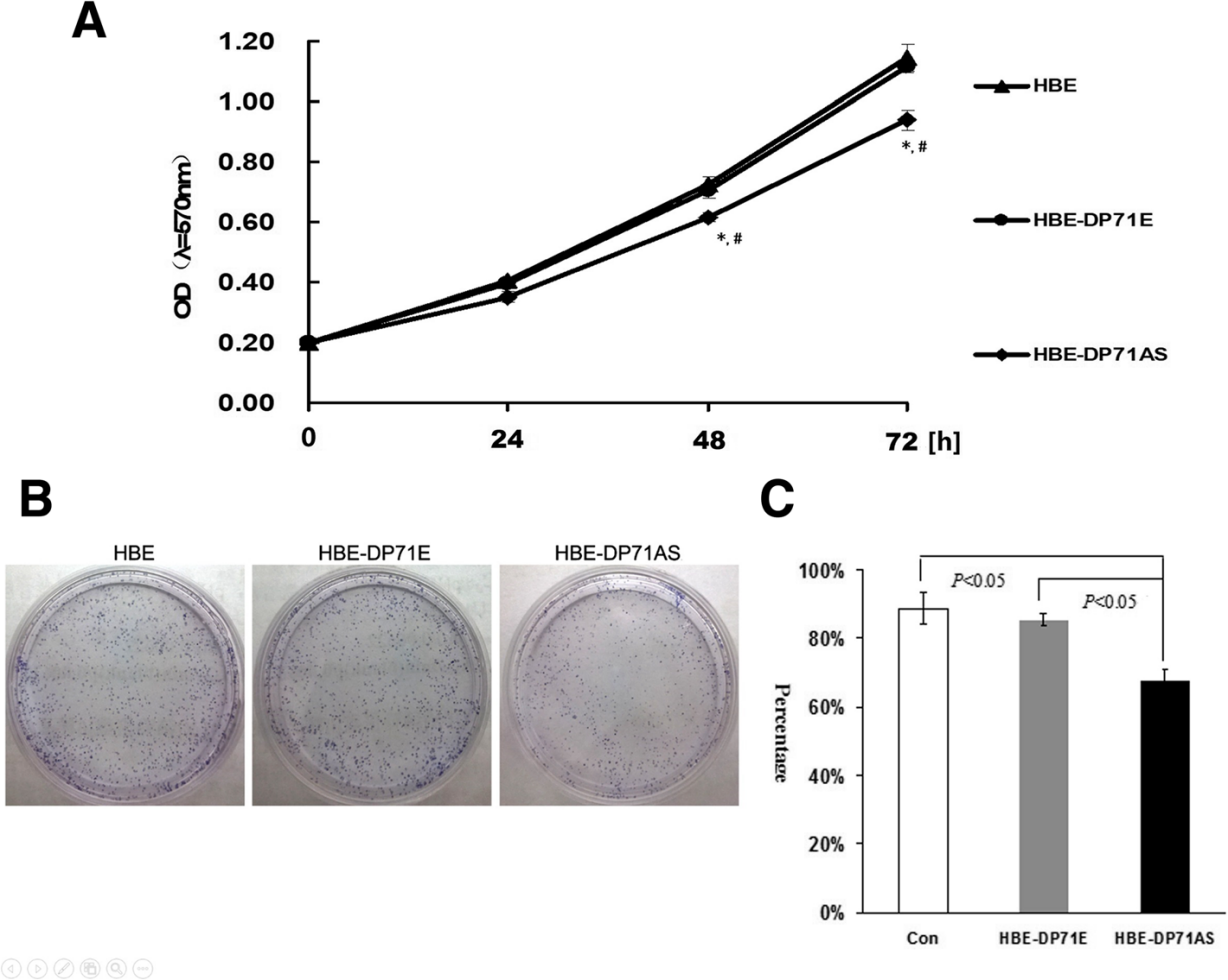
HBE-Dp71AS cells displayed inhibited proliferation. **a** MTT assay was performed to measure the cell growth of HBE-Dp71AS, HBE and HBE-Dp71E cells. The HBE-Dp71AS cells displayed inhibited growth in 24 h, 48 h and 72 h compared with HBE and HBE-Dp71E cells. The differences in 48 h and 72 h are statistically significant,* versus HBE, # versus HBE-Dp71E. **b** Representative photos of clone formation of HBE-Dp71AS, HBE and HBE-Dp71E cells. It is demonstrated that the clonogenic potentials of HBE-Dp71AS were smaller than those of the HBE and HBE-Dp71E cells. Statistical analysis (**c**) proved that the differences are significant

MTT assays were employed to analyze the proliferation of HBE-Dp71AS, HBE-Dp71E and HBE cell groups. Significant growth inhibition was observed at 48 and 72 h in HBE-Dp71AS cells (Fig. 4a), while there were no significant differences in cell growth between HBE-Dp71E and HBE cells (*P* > 0.05). Thus the assays indicated that the ablation of Dp71 protein in HBE cells can effectively inhibit their growth.

### HBE-Dp71 AS cells displayed reduced clone formation capabilities

The anti-cell proliferation effect of Dp71 was assessed by a clonogenic formation assay. There was a significant reduction in the number and size of foci in HBE-Dp71 AS cells compared with HBE-Dp71E and HBE cells (Fig. 4b). The clone formation efficiency for HBE-Dp71E, HBE and HBE-Dp71AS cells was 85.33 ± 1.72, 88.73 ± 4.67 and 67.53% ± 3.45% respectively. The clone formation efficiency for HBE-Dp71AS cells was 20% less than that in HBE-Dp71E and HBE cells; the differences were statistically significant (Fig. 4c). Together with the results from the MTT assay, it is found that ablation of Dp71 protein can significantly inhibit the growth of HBE cells.

### RAD51 interact with Dp71 and Lamin B1 in HBE

**Fig. 5:**
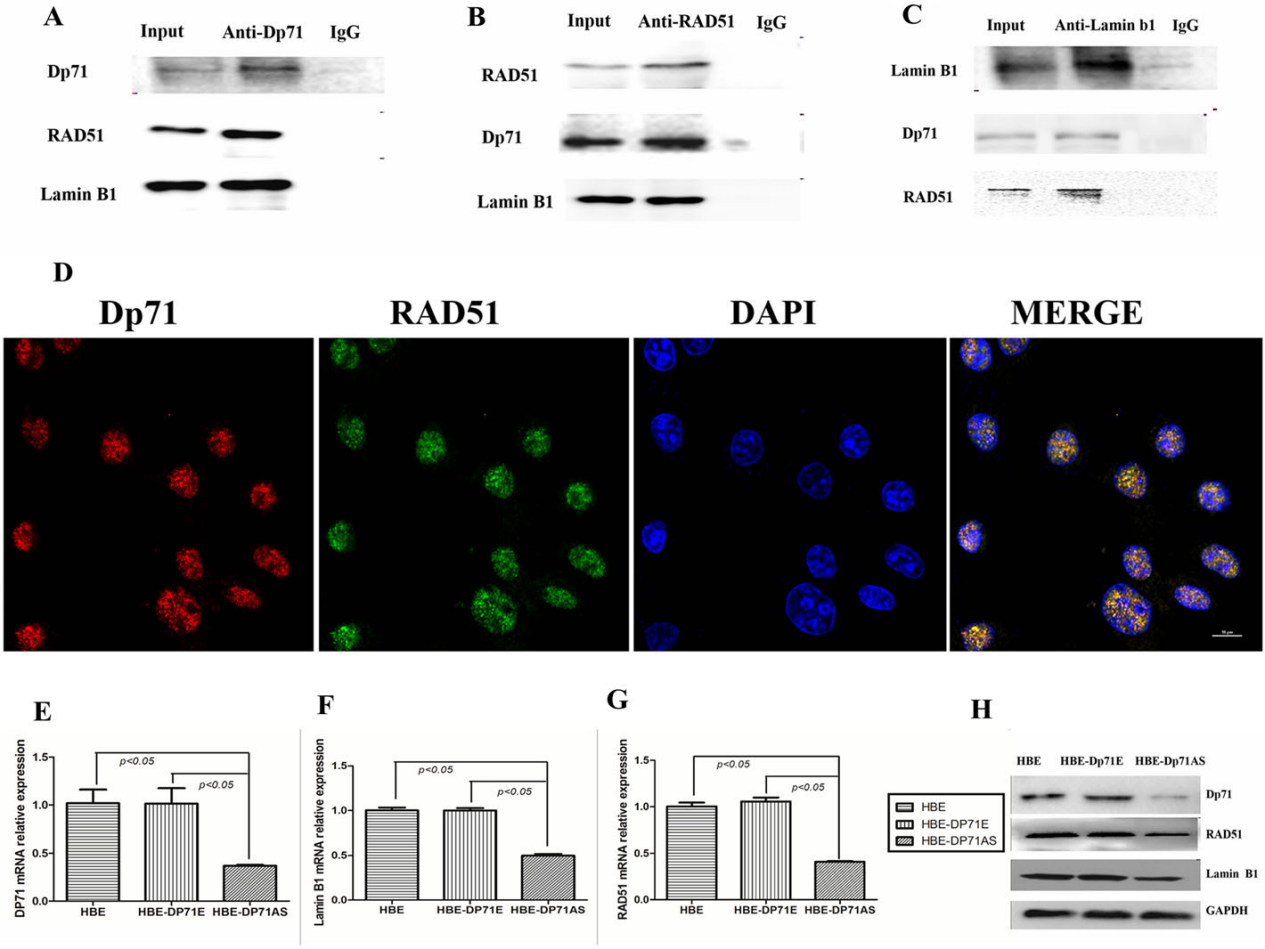
Dp71 interacts with RAD51 in HBE cells. **a** Co-Ip revealed that RAD51 and lamin B1 were successfully pulled down by specific Dp71 antibody, while irrelevant IgG failed to pull down these two proteins. **b** Co-Ip revealed that Dp71 and lamin B1 were successfully pulled down by specific RAD51 antibody, while irrelevant IgG failed to pull down these two proteins. **c** Co-Ip revealed that Dp71 and RAD51 were successfully pulled down by specific lamin B1 antibody, while irrelevant IgG failed to pull down these two proteins. **d** Immunofluorescence displayed the co-localization of Dp71 and RAD51 in HBE cells (scale bar: 10 μm). **e** Significant reduction of Dp71 mRNA was observed in HBE-AS cells. **f** Significant reduction of RAD51 mRNA was observed in HBE-AS cells. **g** Significant reduction of lamin B1 mRNA was observed in HBE-AS cells. **h** Significant reduction of Dp71, RAD51 and lamin B1 protein was observed in HBE-AS cells

RAD51 is a protein that forms discrete nuclear foci and participates in homologous recombination repair upon DNA damage. Interaction between RAD51 and lamin B1 was verified in previous research [9, 10]. In our further characterization of Dp71 in HBE cells, it was found that the Dp71 specific antibody pulled down RAD51 successfully, while non-specific IgG failed to pull down RAD51 (Fig. 5a). With RAD51 antibody, Dp71 was successfully precipitated while IgG failed to work (Fig. 5b). Immunofluorescence verified the interaction between RAD51 and Dp71 in the nucleus (Fig. 5d) and cytoplasm (Additional file 1), while the major interaction occurred in the nucleus. Co-Ip results also proved the association between RAD51 and lamin B1. As indicated in Fig. 5b and c, lamin B1 antibody successfully dragged down RAD51, and RAD51 specific antibody precipitated lamin B1. In each assay, however, non-specific IgG failed to precipitate RAD51 and lamin B1. Combined with the proof of RAD51-Dp71 interaction, we proved the existence of Dp71-RAD51-lamin B1 complex in HBE.

Immunoblot was carried out to quantify lamin B1 and RAD51 expression in HBE-Dp71AS cells. Compared with parental HBE cells and HBE-Dp71E cells, significant reduction of Dp71, lamin B1, RAD51 mRNA (Fig. 5e, f, and g) and protein (Fig. 5h) was observed. The endogenous RAD51 and lamin B1 expression levels were both reduced after the Dp71 expression was knocked down in HBE cells.

### Dp71 depletion resulted in decreased FAK, p-FAK and p-AKT

**Fig. 6:**
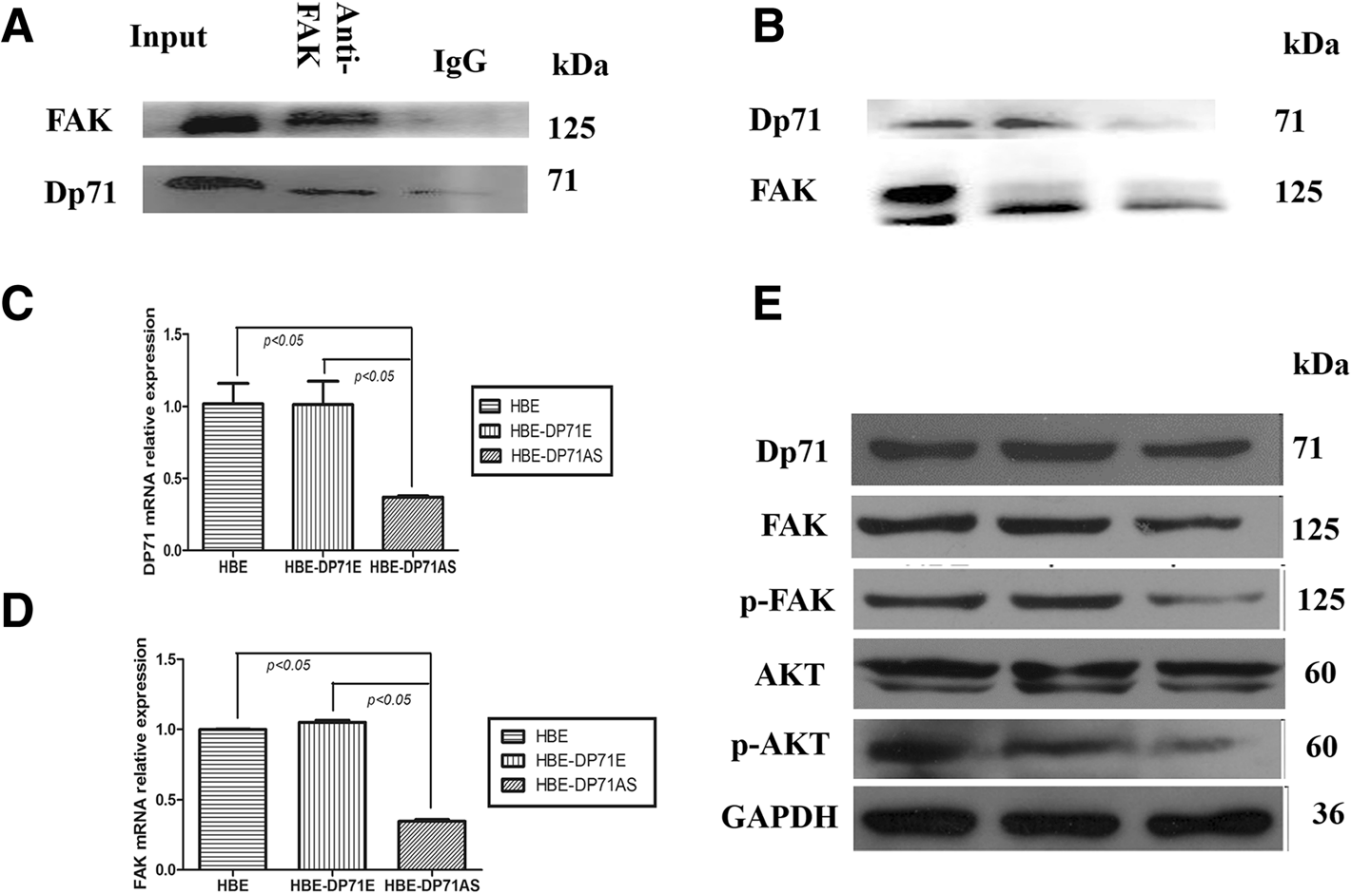
Dp71 depletion resulted in reduced FAK, p-FAK, p-AKT in HBE cells. **a** Co-Ip revealed that FAK was successfully pulled down by specific Dp71 antibody, while irrelevant IgG failed to pull down these two proteins. **b** Co-Ip revealed that Dp71 was successfully pulled down by specific FAK antibody, while irrelevant IgG failed to pull down these two proteins. **c** Significant reduction of Dp71 mRNA was observed in HBE-AS cells. **d** Significant reduction of FAK mRNA was observed in HBE-AS cells. **e** Significant reductions of FAK, p-FAK and p-AKT protein were observed in HBE-AS cells

Dp71 was found associating with most of the β1-integrin complex components (β1-integrin, focal adhesion kinase(FAK), α-actinin, talin and actin) in PC12 cells [11]. Our previous publications proved that FAK is an indispensable component of the cytoplasmic DAPCs in HBE. Via two-way precipitation, Fig. 6a and b show that FAK and Dp71 antibody dragged down each other in the immuno-precipitation process while IgG did not precipitate either protein. The co-Ip assay proved that the cytoplasmic association between Dp71 and FAK is a universal phenomenon in different types of cells.

QRT-PCR displayed reduced FAK mRNA in HBE-Dp71AS cells (Fig. 6c, d). Decreased FAK protein and p-FAK expression was also displayed by immuno-blotting in HBE-Dp71AS cells. Although AKT did not display any expression alteration, p-AKT displayed a significant reduction in HBE-Dp71AS cells (Fig. 6e). Knocking down Dp71 in HBE cells resulted in the reduction of cytoplasmic expression of FAK, p-FAK and p-AKT.

## Discussion

Being one of the most abundantly expressed dystrophin isoforms, Dp71 is distributed in all tissues except skeletal muscle. The deficiency of Dp71 is involved in mental retardation and retinal dysfunction of DMD patients [2, 12, 13]. For years, PC12 cells have been used as the traditional cell model to study the biological function of Dp71. In our further characterization of this ubiquitously expressed scaffolding protein, Dp71 was found to contain more functional diversity than expected. Being one of the newly identified tumor suppressive genes in gastric cancer, our preliminary work identified decreased Dp71 in lung cancer as well. As a usual cell model for pulmonary research, we used shRNA to knock down Dp71 and revealed the biological function of HBE-Dp71AS cells.

After the HBE-Dp71AS cells were stimulated by H_2_O_2_, the alkaline comet assay displayed that tail length and tail moment increased in HBE-Dp71AS cells compared with parental cells and HBE-Dp71E cells. More γ-H2AX foci were detected in the HBE-Dp71AS nucleus. That means more single strand break (SSB) and DSB occurred in HBE-Dp71AS cells under oxidative stress. Knocking Dp71 down increased the DNA damage induced by H_2_O_2._

RAD51 was found to be a new binding partner of Dp71 in our further exploration of HBE-Dp71AS cells. Although cytoplasmic and nuclear interactions between Dp71 and RAD51 were detected in our research, the nuclear Dp71-RAD51 interaction plays a significant role in the increased DNA damage of HBE-Dp71AS cells induced by H_2_O_2_. Co-Ip proved the existence of Dp71-lamin B1-RAD51 protein complex in HBE. RAD51 is proved to be an essential protein for DNA repair by homologous recombination [14, 15]. Overexpression of Rad51 in different organisms and cell types increased homologous recombination (HR) and increased resistance to DNA damaging agents. Being an associating protein of Dp71, lamin B1 has been found to be a binding partner of RAD51, which promotes DSB repair and cell survival by maintaining the RAD51 protein stability [9, 10]. In HBE-Dp71AS cells, depletion of Dp71 results in Rad51, lamin B1 mRNA and protein reduction. RAD51 suffers both decreased expression and impaired stability, which leads to decreased HR and increased DNA damage stimulated by H_2_O_2,_ and finally results in increased apoptosis.

Via interaction with lamin B1, the nuclear Dp71 is proved to affect the cell growth of PC12 and A549. Decreased lamin B1 expression also slows cell proliferation and induces premature senescence in WI-38 cells [6, 16]. In HBE-AS cells, significantly reduced lamin B1 explains the phenotypes of growth inhibition. Increased BRCA1 expression caused by decreased lamin B1 reduction leads to the overactivation of caspase 8 and 3, combined with impaired expression and function of RAD51. The apoptosis rate of HBE-Dp71AS cells increased under H_2_O_2_ stimulation.

**Fig. 7:**
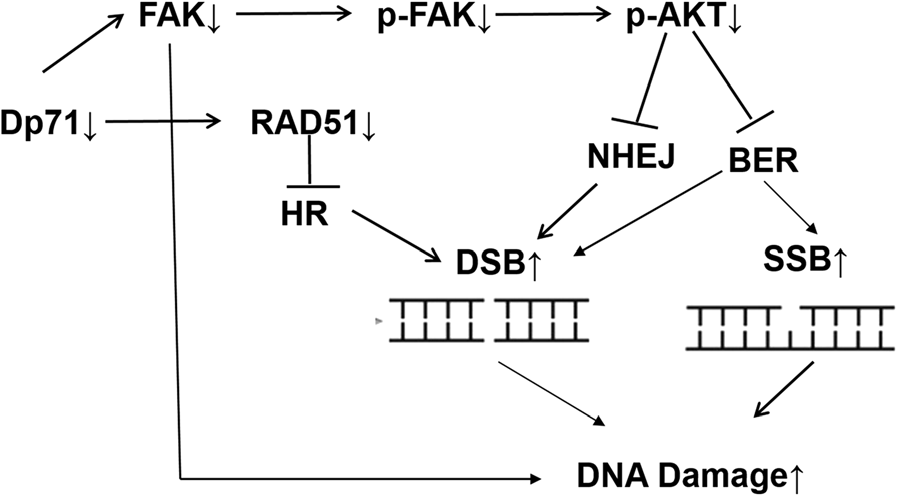
Schematic map on the mechanism of increased DNA damage induced by H_2_O_2_ in HBE-Dp71AS cells. Combined efforts of reduced RAD51, FAK, and p-Akt in HBE-Dp71AS cells finally result in increased DNA damage stimulated by H_2_O_2_

FAK is a cytosolic non-receptor protein tyrosine kinase that regulates cellular adhesion, motility, proliferation and survival in various types of cells [17–19]. FAK is proved to interact with Dp71 in HBE and PC12. Co-Ip also proved the interaction of FAK with Dp71 in HBE in our current research. Decreased FAK mRNA and protein was observed in HBE-Dp71AS cells. FAK is a novel regulator of DNA damage repair in mutant KRAS NSCLC and its pharmacologic inhibition leads to radiosensitizing effects. Specific targeting of focal adhesion kinase in endothelial cells is sufficient to induce tumor-cell sensitization to DNA-damaging therapies and thus inhibit tumor growth in mice. Although FAK-dependent chemo-sensitivity is proved to be related to DNA-damage-induced NF-κB activation, the DNA damage response process FAK participates still needs further exploration. FAK-overexpressed (HL-60/FAK) cells were highly resistant to hydrogen peroxide and ionizing radiation (IR)-induced apoptosis [20, 21]. AKT, also known as protein kinase B, is a serine/threonine-specific protein kinase responsible for apoptosis, cell proliferation, transcription and cell migration. Accumulating evidence has implicated AKT as a direct participant in the DNA damage response and repair induced by commonly used genotoxic agents. AKT plays an important regulatory role in activating DNA-PKcs and non-homologous end joining (NHEJ) repair [22, 23]. Functioning as an activated downstream target in the FAK pathway, AKT protein expression did not show any change in HBE-AS cells. However, the activated AKT was significantly reduced, which acted together with reduced FAK and RAD51 to attenuate the DNA damage of HBE-Dp71AS cells induced by H_2_O_2_ (Fig. 7)_._ Replication errors after this damage would lead to increased mutations and cancer. The reduced FAK and p-AKT can also explain the decreased proliferation of HBE-Dp71AS cells.

For the first time, it is reported that increased DNA damage occurs in Dp71 depleted cells. With the accumulation of studies showing that many of the human hereditary and non- hereditary cancer predisposition diseases are caused by germ-line mutations in DNA repair genes, the pivotal role that DNA repair plays in the in vivo tumorigenesis process becomes evident. We have proved the in vitro reduction of RAD51 and FAK from the transcription level in HBE-Dp71AS cells. RAD51 and FAK are two important proteins participating in DNA damage repair. Such carcinogens as ROS produced by in vivo metabolism attack DNA and cause a variety of DNA lesions. Unrepaired lesions cause gene mutations and chromosomal damage, which may lead to tumor initiation and progression. However, more experimental evidence is needed to prove the role Dp71 plays in the in vivo tumorigenesis.

Being one of the most ubiquitously expressed isoforms of the dystrophin family, our newly revealed biological traits of HBE-Dp71AS cells suggest a much broader role played by Dp71 in many pathophysiological process, which adds some fresh color to this “old” protein.

## Conclusions

Depletion of Dp71, a widely expressed isoform of dystrophin, displays increased DNA damage, decreased proliferation, and increased apoptosis during oxidative stress via decreasing RAD51, lamin B1, pAKT, FAK and pFAK expression.

## Additional file

Additional file 1: Dp71 interacted with RAD51 in HBE cytoplasm. (JPG 1180 kb)

## Data Availability

Additional information related to this study is available from the author for correspondence upon reasonable request.

## Abbreviations

AKT: Protein kinase B
DMD: Duchenne muscular dystrophy
FAK: Focal adhesion kinase
HBE: Human bronchial epithelium
HR: Homologous recombination
NE: Nuclear envelope
NF-κB: Nuclear factor-κB
QRT-PCR: Quantitative real-time polymerase chain reaction
SSB: Single strand break

## References

[1] Jin H, Tan S, Hermanowski J, Bohm S, Pacheco S, McCauley JM (2007). The dystrotelin, dystrophin and dystrobrevin superfamily: new paralogues and old isoforms. BMC Genomics.

[2] Austin RC, Howard PL, D'Souza VN, Klamut HJ, Ray PN (1995). Cloning and characterization of alternatively spliced isoforms of Dp71. Hum Mol Genet.

[3] Bolanos-Jimenez F, Bordais A, Behra M, Strahle U, Sahel J, Rendon A (2001). Dystrophin and Dp71, two products of the DMD gene, show a different pattern of expression during embryonic development in zebrafish. Mech Dev.

[4] Enriquez-Aragon JA, Cerna-Cortes J, Bermudez de Leon M, Garcia-Sierra F, Gonzalez E, Mornet D, Cisneros B (2005). Dystrophin Dp71 in PC12 cell adhesion. Neuroreport..

[5] Aragon J, Romo-Yanez J, Martinez-Herrera A, Ceja V, Rendon A, Montanez C (2011). Characterization of Dp71Delta(78-79), a novel dystrophin mutant that stimulates PC12 cell differentiation. J Neurochem.

[6] Tan S, Tan J, Tan S, Zhao S, Cao X, Chen Z (2016). Decreased Dp71 expression is associated with gastric adenocarcinoma prognosis. Oncotarget..

[7] Tan S, Zhao S, Chen Z, Ma Q, Wang W, Cheng S (2017). Altered biological properties in Dp71 over-expressing HBE cells. Cell Physiol Biochem.

[8] Tan S, Tan S, Chen Z, Cheng K, Chen Z, Wang W (2016). Knocking down Dp71 expression in A549 cells reduces its malignancy in vivo and in vitro. Cancer Investig.

[9] Liu NA, Sun J, Kono K, Horikoshi Y, Ikura T, Tong X (2015). Regulation of homologous recombinational repair by Lamin B1 in radiation-induced DNA damage. FASEB J.

[10] Butin-Israeli V, Adam SA, Jain N, Otte GL, Neems D, Wiesmuller L (2015). Role of Lamin b1 in chromatin instability. Mol Cell Biol.

[11] Cerna J, Osuna-Castro JA, Muniz J, Mornet D, Garcia-Sierra F, Cisneros B (2009). Dystrophin Dp71f associates with components of the beta1-integrin adhesion complex in PC12 cell neurites. Acta Neurol Belg.

[12] Austin RC, Morris GE, Howard PL, Klamut HJ, Ray PN (2000). Expression and synthesis of alternatively spliced variants of Dp71 in adult human brain. Neuromuscul Disord.

[13] Banihani R, Smile S, Yoon G, Dupuis A, Mosleh M, Snider A, McAdam L (2015). Cognitive and neurobehavioral profile in boys with Duchenne muscular dystrophy. J Child Neurol.

[14] Magwood AC, Malysewich MJ, Cealic I, Mundia MM, Knapp J, Baker MD (2013). Endogenous levels of Rad51 and Brca2 are required for homologous recombination and regulated by homeostatic re-balancing. DNA Repair (Amst).

[15] Matsunami K, Otsuka H, Xu H, Firdawes S, Yamamoto A, Ishimaru A, Fukuzawa M, Miyagawa S (2008). Molecular cloning of pig Rad51, Rad52, and Rad54 genes, which are involved in homologous recombination machinery. Transplant Proc.

[16] Villarreal-Silva M, Centeno-Cruz F, Suarez-Sanchez R, Garrido E, Cisneros B (2011). Knockdown of dystrophin Dp71 impairs PC12 cells cycle: localization in the spindle and cytokinesis structures implies a role for Dp71 in cell division. PLoS One.

[17] Chen JS, Huang XH, Wang Q, Chen XL, Fu XH, Tan HX, Zhang LJ, Li W, Bi J (2010). FAK is involved in invasion and metastasis of hepatocellular carcinoma. Clin Exp Metastasis.

[18] Crosara-Alberto DP, Inoue RY, Costa CR (2009). FAK signalling mediates NF-kappaB activation by mechanical stress in cardiac myocytes. Clin Chim Acta.

[19] Owen KA, Abshire MY, Tilghman RW, Casanova JE, Bouton AH (2011). FAK regulates intestinal epithelial cell survival and proliferation during mucosal wound healing. PLoS One.

[20] Tamagiku Y, Sonoda Y, Kunisawa M, Ichikawa D, Murakami Y, Aizu-Yokota E, Kasahara T (2004). Down-regulation of procaspase-8 expression by focal adhesion kinase protects HL-60 cells from TRAIL-induced apoptosis. Biochem Biophys Res Commun.

[21] Huang D, Khoe M, Befekadu M, Chung S, Takata Y, Ilic D, Bryer-Ash M (2007). Focal adhesion kinase mediates cell survival via NF-kappaB and ERK signaling pathways. Am J Physiol Cell Physiol.

[22] Liu Q, Turner KM, Alfred Yung WK, Chen K, Zhang W (2014). Role of AKT signaling in DNA repair and clinical response to cancer therapy. Neuro-Oncology.

[23] Tavora B, Reynolds LE, Batista S, Demircioglu F, Fernandez I, Lechertier T, Lees DM, Wong PP, Alexopoulou A, Elia G, Clear A, Ledoux A, Hunter J, Perkins N, Gribben JG, Hodivala-Dilke KM (2014). Endothelial-cell FAK targeting sensitizes tumours to DNA-damaging therapy. Nature..

