# Supplementary figures and images for: Dp71 depleted HBE cells displayed increased DNA damage and apoptosis induced by H2O2
Source: Cell Mol Biol Lett. 2019 Jun 17;24:42. doi: 10.1186/s11658-019-0169-6 (PMC6580496; doi:10.1186/s11658-019-0169-6)

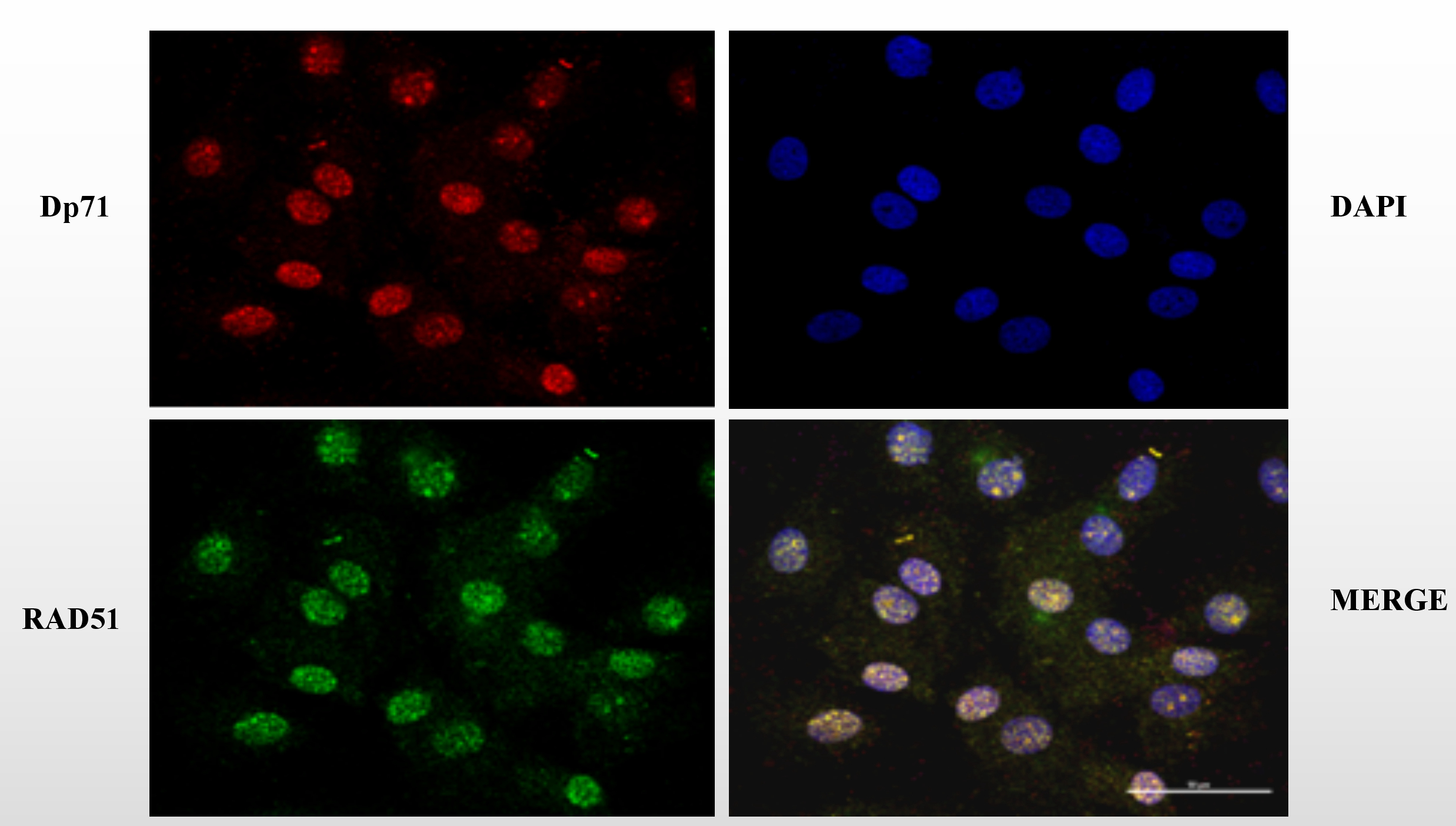

Supplement: Supplementary file 1 — Dp71 interacted with RAD51 in HBE cytoplasm. (JPG 1180 kb) [file 11658_2019_169_MOESM1_ESM.jpg]
